# Combined Free Fibula Osteocutaneous and Anterolateral Thigh-Vastus Lateralis Free Flaps for Clavicule and Extensive Chest Wall Reconstruction After Sarcoma Resection

**DOI:** 10.7759/cureus.8391

**Published:** 2020-06-01

**Authors:** Pedro Ciudad, Maria T Huayllani, Antonio J Forte, Francisco R Avila, Hung-Chi Chen

**Affiliations:** 1 Plastic, Reconstructive and Burn Surgery, Arzobispo Loayza National Hospital, Lima, PER; 2 Plastic Surgery, Mayo Clinic Florida, Jacksonville, USA; 3 Plastic and Reconstructive Surgery, China Medical University Hospital, Taichung, TWN

**Keywords:** case report, clavicle reconstruction, microvascular, vascular reconstruction, chest wall, thoracic malignancy

## Abstract

Combined extensive chest and neck reconstructions is a challenging procedure. The rapid development in microvascular surgery has allowed the cancer surgeons to successfully resect and reconstruct advanced malignancies in the neck and thoracic region. Herein we present a young female diagnosed with malignant mesenchymal sarcoma of the right side of the neck extending to right upper lung and anterior mediastinum. The patient was successfully treated with two microvascular free flaps in a multidisciplinary approach. Wide local resection of the tumor was done along with removal of right upper pulmonary lobe and the subclavian vessels. Vascular reconstruction was done with polytetrafluoroethylene grafts. A free fibula osteocutaneous flap was used for stabilization and reconstruction of the clavicle. Anterolateral thigh flap with vastus lateralis muscle was used for soft tissue reconstruction. All flaps survived and the patient had a good recovery at three months of follow-up. Future reports suggesting guidelines or algorithms for complex chest wall reconstruction should benefit of similar scenarios to the one reported here.

## Introduction

Extensive chest wall resections usually result in considerable morbidity affecting the daily living of patients [1]. Approximately 75% to 90% of chest wall defects are covered with regional or pedicled myocutaneous flaps; however, the type of flap and procedure requires a careful planning and individualized management [2,3]. The rapid development in microvascular surgery has allowed the cancer surgeons to successfully resect and reconstruct advanced malignancies in the neck and thoracic region. In fact, surgical techniques to reconstruct chest wall defects after tumor resection have increased over the time [2,4]. However, a few reports have attempted to classify and present a protocol for the management of chest wall defects [3,5,6]. Herein, we aim to present a case of an extensive chest wall reconstruction after a sarcoma resection using polytetrafluoroethylene (PTFE) grafts for subclavian vessels, fibula osteocutaneous flap for clavicle reconstruction, and the anterolateral thigh-vastus lateralis flap to cover the soft tissue defect. The report attempts to bring focus on the complexities encountered in chest wall reconstruction.

## Case presentation

**Figure 1:**
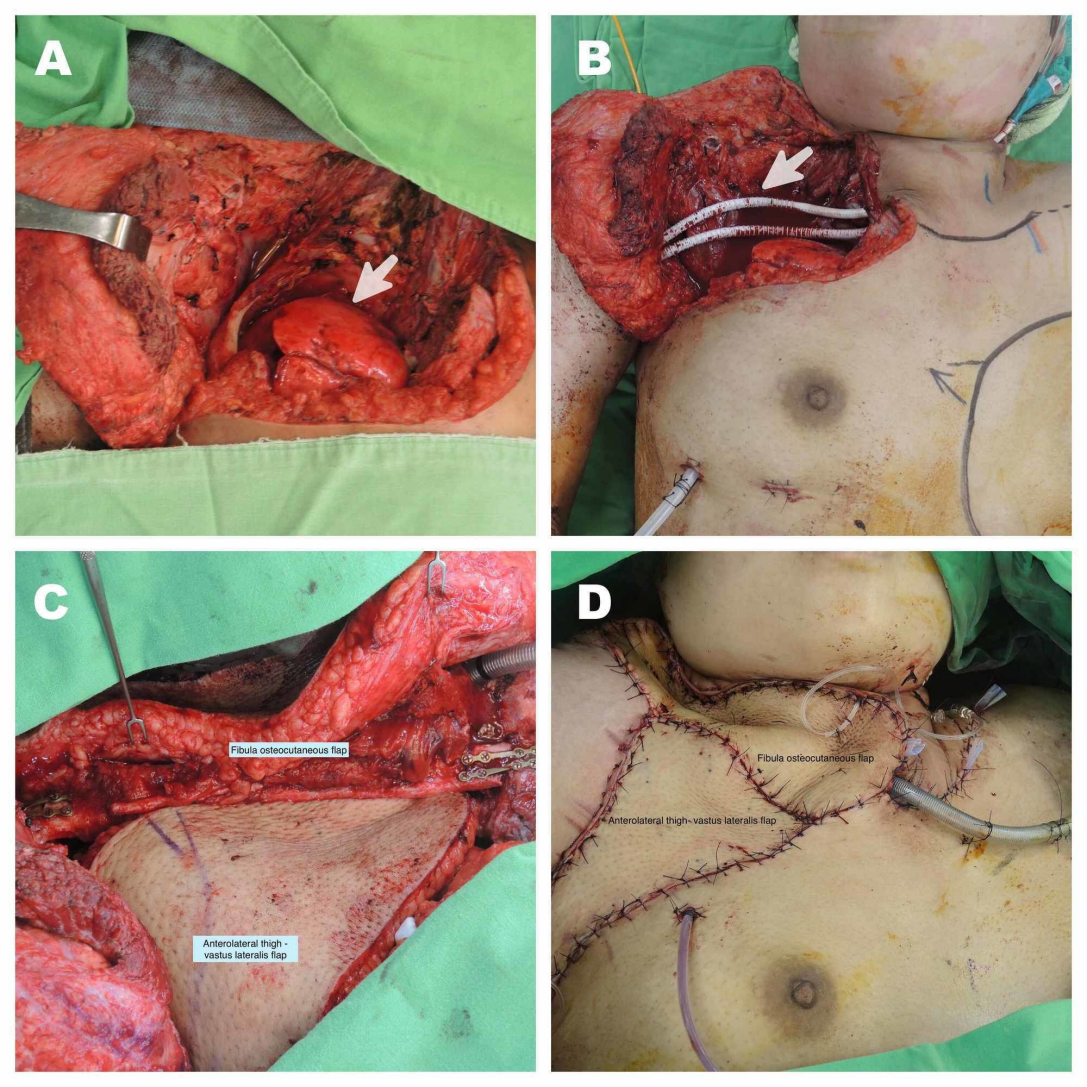
Complex chest wall reconstruction. (A) Chest wall defect after resection of the sarcoma. Absence of right clavicle and four first ribs, arrow shows the superior lobe of the lung. (B) Subclavian vessels reconstruction (arrow). (C) Clavicle reconstruction with fibula osteocutaneous flap and anterolateral thigh-vastus lateralis flap. (D) Immediate postoperative reconstruction.

A 24-year-old female was referred at our center with the diagnosis of malignant mesenchymal sarcoma of the right side of the neck that extends to right upper lung and anterior mediastinum. After careful evaluation, the oncosurgical team planned a wide local resection. The soft tissue sarcoma was removed with a 2 cm margin along with the upper lobe of the right lung. The first four ribs and the clavicle of the affected side were also removed (Figure 1).

**Figure 2:**
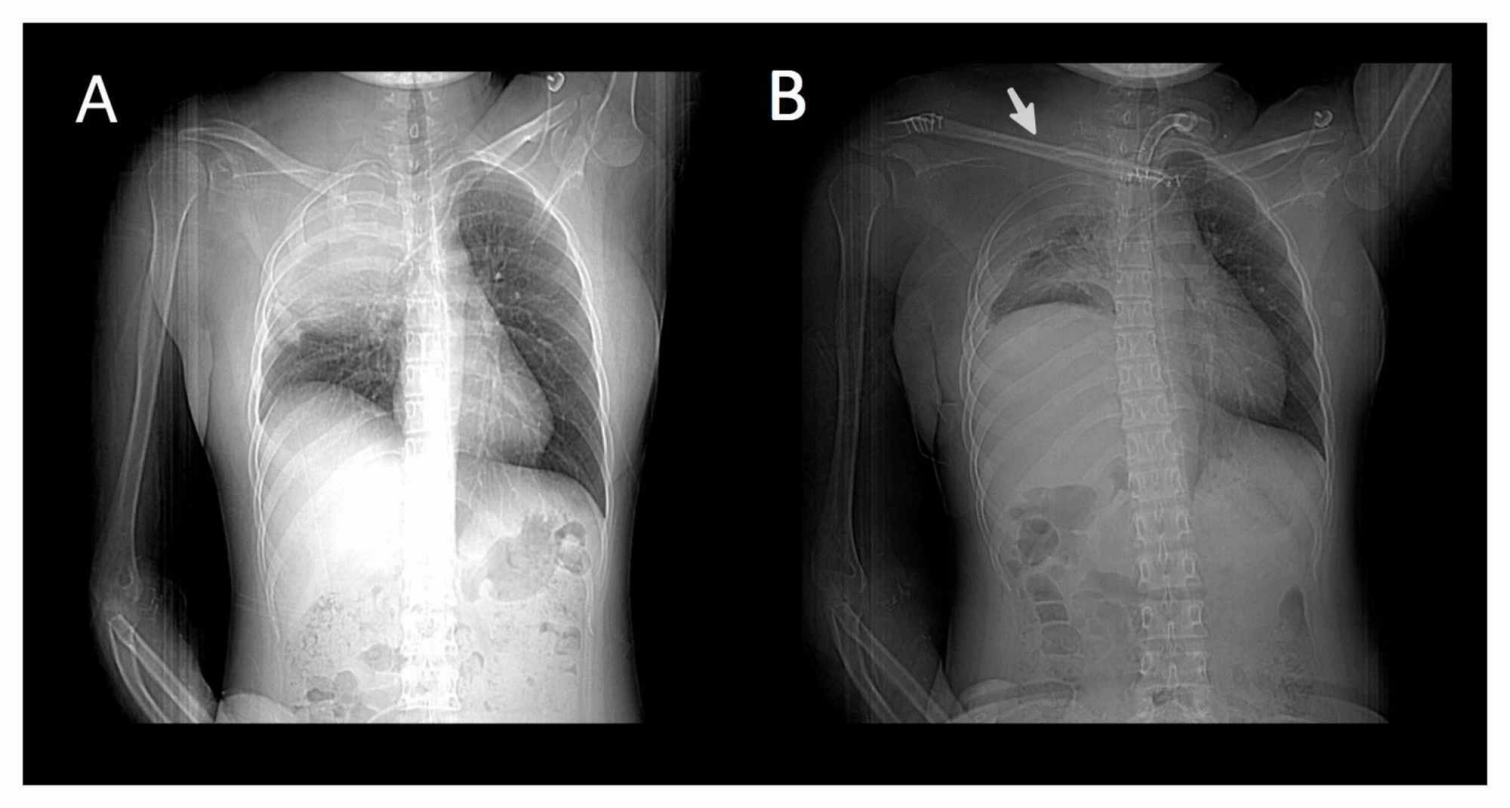
Chest X-ray. (A) Preoperative and (B) postoperative, arrow shows fibula osteocutaneous flap.

A vascular team reconstructed the subclavian artery and vein using PTFE grafts. Then, the reconstructive team planned a fibula osteocutaneous flap for clavicle stabilization, reconstruction, and resurfacing of the neck and thorax region. The fibula was fixed with miniplates, medially to the sternum and laterally to the remnant of the right clavicle. The flap was vascularized by the anastomosis of superior thyroid artery with the peroneal artery, along with a venous drainage from the peroneal comitant veins to the left external jugular vein. In both anastomoses, an interposed vein graft was utilized. The ischemia time was 80 minutes. In order to obliterate the pulmonary dead space and to provide coverage of the PTFE grafts and thoracic resurfacing, an anterolateral thigh flap measuring 25 cm x 12 cm was harvested along with the left vastus lateralis muscle. The descending branch of lateral circumflex femoral artery and the right thoracodorsal artery were anastomosed in an end-to-end fashion. The venous drainage ran from the comitant veins of the descending branch of lateral circumflex femoral to the thoracodorsal pedicle. The ischemia time was 70 minutes. The donor sites were resurfaced with 10/1,000 inch skin thickness skin graft. The patient stood the operation well. Both flaps survived completely. There were no donor site complications. Radiogram at three months of follow-up showed clavicle reconstruction with the transferred fibula flap (Figure 2). The patient was followed up by cardiovascular surgery, pulmonology, and physical therapy specialists with good recovery at three months post-surgery.

## Discussion

Combined extensive chest and neck reconstruction is a challenging procedure that requires a multidisciplinary approach regardless of the origin and pathology [3]. In general, reconstruction is indicated when a defect is more than 5 cm in diameter or includes more or equal four ribs to avoid the risk of lung herniation and chest wall instability that compromises the respiration [7,8]. Multiple free flaps may be indicated for the complex requirement of the region. The situation may be further complicated when there is underlying vascular reconstruction with synthetic PTFE grafts. These two aspects mandated a watertight soft tissue cover to avoid graft sepsis and complications like vascular blowout. The fibula flap was indicated to neutralize the chest wall instability caused due to the removal of the clavicle and the first four ribs. It also provided protection to the underlying PTFE grafts, whereas the anterolateral thigh-vastus lateralis flap allowed to fill up the dead space left after resection of the pulmonary lobe. The osteocutaneous fibula flap is considered the standard care for reconstruction of mandibular defects with a high rate of success [9,10]. Moreover, it can also be effective to reconstruct long bone defects after sarcoma resections [11]. Although it has been reported that this flap is also successful for sternoclavicular joint defects following resection of tubercular osteomyelitis, no reports were found to evaluate its efficacy for sarcomas [12]. Our case supports the use of the fibula osteocutaneous flap for clavicular defects after sarcoma resection and anterolateral thigh-vastus lateralis flap to cover the remaining defect in complex chest reconstructions.

Although we are reporting only a single case, the successful outcome suggests that even with advanced malignancy and aggressive excision, optimal reconstruction can still be achieved with an adequate plan and microsurgical skills. A hypothesis can be built up only after implementing the management strategy in larger series.

## Conclusions

This report highlights the complexities involved in chest wall reconstruction. Although clavicle reconstruction is not usually indicated, it becomes important in combined neck and chest reconstructions, especially when vascular reconstruction has also been done. Future reports suggesting guidelines or algorithms for chest wall reconstruction should benefit of similar scenarios to the one reported here.
